# Impact of the gut microbiome composition on social decision-making

**DOI:** 10.1093/pnasnexus/pgae166

**Published:** 2024-05-14

**Authors:** Marie Falkenstein, Marie-Christine Simon, Aakash Mantri, Bernd Weber, Leonie Koban, Hilke Plassmann

**Affiliations:** Control-Interoception-Attention Team, Sorbonne Université, Paris Brain Institute (ICM), Inserm, CNRS, APHP, Hôpital de la Pitié Salpêtrière, 47 boulevard de l’Hôpital, 75013 Paris, France; Institute of Nutrition and Food Science (IEL), Nutrition and Microbiota, University of Bonn, Katzenburgweg 7, 53115 Bonn, Germany; Institute of Nutrition and Food Science (IEL), Nutrition and Microbiota, University of Bonn, Katzenburgweg 7, 53115 Bonn, Germany; Institute for Genomic Statistics and Bioinformatics, University of Bonn and University Hospital Bonn, Venusberg Campus 1, 53127 Bonn, Germany; Institute of Experimental Epileptology and Cognition Research, University of Bonn and University Hospital Bonn, Venusberg-Campus 1, 53127 Bonn, Germany; Control-Interoception-Attention Team, Sorbonne Université, Paris Brain Institute (ICM), Inserm, CNRS, APHP, Hôpital de la Pitié Salpêtrière, 47 boulevard de l’Hôpital, 75013 Paris, France; Marketing Area INSEAD, Boulevard de Constance, 77300 Fontainebleau, France; Lyon Neuroscience Research Center, CNRS, INSERM, Claude Bernard University Lyon 1, CH Le Vinatier - Bâtiment 462 - Neurocampus, 95 Bd Pinel, 69500 Bron, France; Control-Interoception-Attention Team, Sorbonne Université, Paris Brain Institute (ICM), Inserm, CNRS, APHP, Hôpital de la Pitié Salpêtrière, 47 boulevard de l’Hôpital, 75013 Paris, France; Marketing Area INSEAD, Boulevard de Constance, 77300 Fontainebleau, France

**Keywords:** ultimatum game, microbiome, social decision-making, gut–brain axis

## Abstract

There is increasing evidence for the role of the gut microbiome in the regulation of socio-affective behavior in animals and clinical conditions. However, whether and how the composition of the gut microbiome may influence social decision-making in health remains unknown. Here, we tested the causal effects of a 7-week synbiotic (vs. placebo) dietary intervention on altruistic social punishment behavior in an ultimatum game. Results showed that the intervention increased participants’ willingness to forgo a monetary payoff when treated unfairly. This change in social decision-making was related to changes in fasting-state serum levels of the dopamine-precursor tyrosine proposing a potential mechanistic link along the gut–microbiota–brain-behavior axis. These results improve our understanding of the bidirectional role body–brain interactions play in social decision-making and why humans at times act “irrationally” according to standard economic theory.

**Significance Statement**

The composition of the gut microbiome can influence behavior and health. However, little is known about how the gut microbiome may impact human social decision-making. We show that a dietary intervention changed the composition of the gut microbiome, which in turn changed people's decisions in a standard social dilemma: Fairness became more important when deciding to accept or reject different monetary payoffs. Our results provide causal evidence for effects of the gut microbiome composition on social decision-making and point to a role of the dopamine-precursor tyrosine. They provide new insights on the role of the microbiome–gut–brain axis for social behavior and highlight the importance of a balanced diet for social behavior, with potential implications for education and policy.

## Introduction

Gut and brain are known to interact in bidirectional and complex ways. An exciting recent research stream in biological and neural science is how the gut microbiome the microorganisms residing in the gastrointestinal tract impacts behavior, cognition, and brain function in its host (1, 2). In particular, social behavior seems to be tightly linked to gut–brain interactions (3). Social interactions shape the composition of the microbiome, but the microbiome may also modulate social behavior. A growing body of research has linked the diversity and composition of the gut microbiome to socio-affective behavior in animal models as well as clinical conditions (3–6). For example, previous animal research has found that germ-free mice exhibit social impairments and a reduction in anxiety-like behavior (7–11). Implanting the microbiota of humans with autism spectrum disorder in germ-free mice led them to display autism-like behaviors (12), and implanting the feces of dysbiotic alcohol-dependent patients induced social impairments in mice, mirroring the affective and social difficulties observed in these patients (6). In humans, a recent study found the alpha diversity of the gut microbiome (i.e. the diversity of different microbes within one sample [13]) to be lower in patients with depressive symptoms (14) and the beta-diversity of the gut microbiome (i.e. the diversity of different microbes between two samples [13]) to be significantly greater between patients with autism spectrum disorder (8, 15). Together, all of this research suggests that the gut microbiome might play a modulatory role in social cognition and behavior. Against this background, the goal of this research was to (i) investigate the effects of human gut microbiome composition on social decision-making and (ii) shed light on the underlying mechanisms along the microbiota–gut–brain axis.

Social decision-making refers to processes in which individuals make choices within a social context, meaning that their decisions have consequences for both the individual and others (16). A large body of evidence shows that social decisions are not influenced solely by self-interest but also by social norms such as fairness considerations. For example, the concept of altruistic punishment describes forgoing personal interest to penalize behavior that is not in line with social norms (17).

Altruistic punishment is typically studied in the ultimatum game (UG) a classic task from behavioral economics (18) in which humans often reject monetary payoffs when receiving an unfair offer. In more detail, one participant (the proposer) is endowed with a specific amount of money (e.g. €10) and offers a share of the money to a second participant (the responder), who can either accept or reject the offer. If the responder rejects the offer, neither participant receives any money. When participants play this game only once and with an anonymous other participant, it should be their economically rational choice to accept any offer greater than €0. However, many studies (19) show that offers greater than €0 but perceived as unfair (such as €1 or €2 out of €10) are typically rejected reflecting altruistic punishment. Here, we tested whether the behavior of responders in the UG was altered by a 7-week synbiotic intervention.

Human and animal models suggest that gut microbiota can communicate with the central nervous system via several different pathways (20–22). Besides signaling via the vagus nerve, gut-related information can reach the brain by means of biochemical signals, such as those via the immune system, microbial metabolites, gut peptides, and neurotransmitters (3, 22). For example, the microbiota–gut–brain axis is involved in the release and metabolism of dopamine and serotonin and their precursors (23, 24). These precursors include large neutral amino acids (LNAA) such as tyrosine and tryptophan precursors for dopamine and serotonin, respectively (25) that can modulate (social) cognition and brain function (26).

Further, LNAA seem to be involved in social decision-making processes such as trust, generosity, and antisocial and altruistic behavior (27, 28): For instance, after administration of the serotonin precursor tryptophan, behavior seen as immoral was judged as more reprehensible (29). Social discounting (i.e. the decrease in generosity as social distance to others increases) decreased after the receipt of a dopamine agonist, with a significant reduction in generous behavior especially to socially close others (30). Specifically, fairness-related social decision-making and altruistic punishment was shown to be sensitive to different states of dopamine and serotonin (31, 32). For example, a dietary intervention causing tryptophan depletion via an amino acid drink led to more altruistic punishment (i.e. rejection) of unfair offers in a UG (31, 33). Another study manipulated a breakfast by either increasing or decreasing the carbohydrate/protein ratio, which increases tryptophan or tyrosine respectively. This diet-induced decrease in tyrosine (but not in tryptophan) led to an increase in rejection rates of unfair offers (34).

**Fig. 1.**
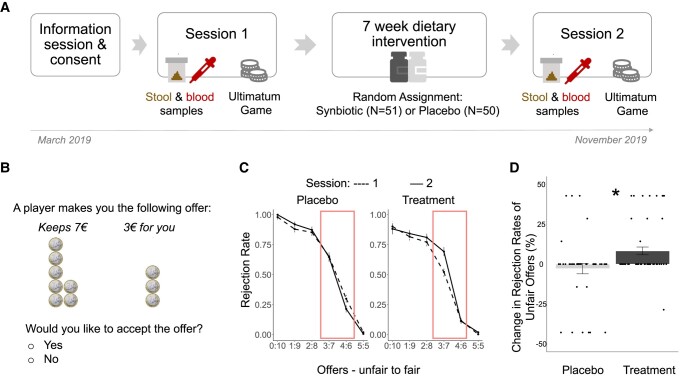
A) Study flow and randomization. B) Sample trial of an unfair offer in the ultimatum game. C) Distribution of rejection rates of all offers for each group and each session. D) Change in rejection rates of unfair offers across sessions for each group (to improve visibility, points are jittered). Error bars represent the standard error of the mean; *P < 0.05.

In this research, we integrated the literature on interactions between socio-affective processes and the gut microbiome with recent evidence that diet may affect social decision-making. We investigated (i) whether a change in the gut microbiome composition changed altruistic punishments, measured as rejection rates in the UG and (ii) whether this effect can be linked to changes in circulating tyrosine and tryptophan levels. In a preregistered (https://osf.io/utsn4) randomized, double-blind, placebo-controlled study, we altered the gut microbiome composition in human adults temporarily through a subtle dietary intervention by administering a commercially available pro- and prebiotic (synbiotic) supplement, without changing the macronutrient (i.e. carbohydrate, protein, and fat) composition of the participants’ diets^a^. We compared the effects of the synbiotic dietary supplement in the intervention group (*n* = 51) with the effects on the participants in a matched placebo control group (*n* = 50; Fig. 1A and Table S1 show group characteristics and lack of difference at baseline).

We chose the administration of a synbiotic supplement for several reasons: First, previous work has shown that the gut microbiome diversity and composition is shaped by among other environmental factors what we eat (35) and can be improved by a change in the host's diet (36, 37). Second, we aimed to increase the diversity of participants’ gut microbiome composition after vs. before the intervention by (i) providing them with beneficial bacteria (i.e. *Lactobacillus* and *Bifidobacterium* [38, 39]) and (ii) increasing the beneficial bacteria already present in the intestine by administering the prebiotic inulin to increase the probability that those bacteria would colonize (40). Third, our goal was to test whether a subtle, ecologically common change in diet the intake of a commercially available supplement in addition to one's regular diet could change the gut microbiome composition and in turn social decision-making.

Our study's participants provided stool and blood samples before and after the 7-week dietary intervention, allowing us to measure gut microbiome composition and diversity as well as levels of circulating LNAAs (see Fig. 1A). They played a one-shot UG to assess their tendency to inflict altruistic punishment in the form of rejection rates before and after the intervention (see Fig. 1B). We preregistered the hypotheses that (i) the intake of the dietary supplement would alter the composition and diversity of the gut microbiome and (ii) the change in the gut microbiome composition and diversity would impact the human metabolism and thereby decision-making behavior. More specifically, we preregistered to explore whether changes in the gut microbiome diversity would change LNAA plasma levels (e.g. tyrosine and tryptophan) and whether this change could be linked to changes in rejection rates in the UG^b^.

## Results

We first tested whether the intake of the synbiotic supplement changed the rejection rates in the UG. Using a generalized linear mixed-effects model, we tested the effects of group, time, and their interaction on the rejection of offers differing in amount (and thereby fairness). We included participants and trials as random effects. We found a significant effect of group (β_Group_ = −1.86, SE = 0.78, *P* = 0.017) and a significant group × time interaction (β_GroupxSession_ = 1.13, SE = 0.28, *P* < 0.001): Participants in the treatment group rejected a higher proportion of offers after the intake of the synbiotic treatment, thus showing an increased tendency for altruistic punishments (see Fig. 1C and D and Table S3, model 1). These results were robust when controlling for age and body mass index (BMI; see Table S3, model 2), and for age and metabolic health (see Table S3, model 3).

Despite our random group assignment, there were differences between the groups before the intervention. Subsequent analyses assessed whether changes in rejection rates were significantly different from zero in each experimental group. Wilcoxon signed-rank tests yielded a significant difference for the treatment group in changes in rejection of all offers (W statistic = 178, *P* = 0.031) but not for the placebo group (W statistic = 142, *P* = 0.406). These findings suggest that despite baseline differences, the synbiotic but not the placebo increased rejection of offers.

The effect was driven by changes in rejection of unfair (30–40% split) but not very unfair (less than 30% split; see Fig. 1C) offers, in line with previous findings that people usually accept fair offers (50% or more split) and reject very unfair offers (19, 31–34). We used a linear regression analysis to examine the impact of group on the change in rejection of unfair offers over time. We found a significant group effect (β_Group_ = 0.43, SE = 0.28, *P* = 0.032; see Fig. 1B), implying that the treatment group experienced a higher change in the rejection of unfair offers compared to the control group (see Table S4). Against this background, all subsequent analyses that aimed at understanding the underlying processes of this effect used the change of rejection of unfair offers over time as a dependent variable.

**Fig. 2.**
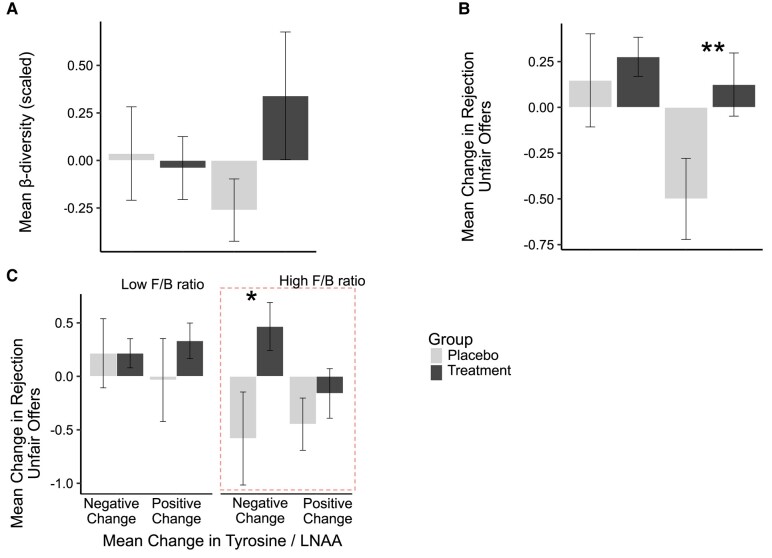
Intervention effects depend on the homeostatic balance of gut microbiome composition (i.e. the F/B ratio). For visualization purposes, we illustrate the continuous F/B ratio as a categorical variable using a median split: A) the β-diversity reflecting the change in gut microbiome composition over time is significantly different between treatment groups depending on their F/B ratio. The higher the baseline F/B ratio the greater the impact of the intervention on β-diversity. B) The mean change in rejection of unfair offers is higher for participants in the treatment group as compared to the placebo group if they had a higher baseline F/B ratio as revealed by post hoc *t*-tests (*t*(1971.7) = 2.73, *P* = 0.006). C) Participants in the treatment group with a higher F/B ratio and a negative change in tyrosine/LNAA rejected more unfair offers than participants whose change was positive as revealed by post hoc *t*-tests (*t*(14.849) = −2.14, *P* = 0.049). Error bars represent the standard error of the mean; **P < 0.01, *P < 0.05.

We next tested our hypothesis that the 7-week daily intake of the synbiotic dietary supplement (as compared to a placebo) changed the composition of the gut microbiome. The difference in composition between baseline and postintervention was measured as the beta-diversity between the first and the second microbiome samples’ composition. We found that participants’ beta-diversity (i.e. change in gut microbiome composition) depended on the balance of their gut microbiome composition at baseline as captured by the Firmicutes to Bacteroidetes ratio (F/B ratio): Having a balanced ratio of the two most common phyla (i.e. Firmicutes and Bacteroidetes (3)) is crucial for homeostasis of the gut (41). For example, a high F/B ratio has been linked to a more animal protein-based Western diet (42) and to obesity (43). In addition, this ratio is linked to the dietary supplement we administered as it included beneficial probiotics in the Firmicutes phylum (44) and inulin, which increases the abundance of Bifidobacteria while decreasing Bacteroides (40). We found that the higher the F/B ratio at baseline, the higher the change in the gut microbiome composition over time in the intervention group (β_GroupxF/B ratio_ = 0.78, SE = 0.35, *P* = 0.029; see Fig. 2A and Table S5). Thus, the intervention had a greater impact on participants with an unbalanced intestinal microbiome before the intervention.

Because the strength of the effect of the intervention on the microbiome composition was dependent on the level of intestinal homeostasis at baseline (i.e. the F/B ratio), we next explored whether the same moderation was at play for the effect of the synbiotic intervention on the change in rejection rates of unfair offers. We found that indeed, participants with a higher F/B ratio at baseline who received the synbiotic supplement had a higher increase in rejection of unfair offers after the intervention compared to the placebo group (β_Group_ = 0.54, SE = 0.19, *P* = 0.006; β_F/B ratio_ = −0.97, SE = 0.33, *P* = 0.005; β_GroupxF/Bratio_ = 0.89, SE = 0.35, *P* = 0.012; see Table S4, model 4, and Fig. 2B; post hoc *t*-tests in the high F/B ratio group: *t*(1971.7) = 2.73, *P* = 0.006).

Finally, we explored the mechanisms underlying the effects of this intervention on participants’ altruistic punishment behavior by linking it to previous findings on the role of dopamine and serotonin precursors for altruistic punishment (31, 33, 34). To this end, we tested whether our intervention changed plasma levels of the dopamine-precursor tyrosine and the serotonin precursor tryptophan. We found an overall trend that tyrosine plasma levels increased after the intake of the synbiotic supplement and that this relationship again depended on the baseline homeostatic balance of the gut microbiome (i.e. the F/B ratio; β_Group_ = 0.09, SE = 0.20, *P* = 0.642; β_F/B ratio_ = 0.60, SE = 0.34, *P* = 0.086; and β_GroupxF/B ratio_ = −0.81, SE = 0.36, *P* = 0.026; see Table S6). No such effects were found for tryptophan (see Table S6).

Given the effect of our intervention on tyrosine levels, we then explored whether intervention-induced changes in tyrosine were linked to the impact of the intervention on changes in altruistic punishment. Indeed, we found that rejection rates for unfair offers increased for participants with a high F/B ratio and a reduction in tyrosine in the treatment group but not in the placebo group (β_Group × F/B ratio × Tyrosine/LNAA ratio_ = −2.19, SE = 0.97, *P* = 0.027; see Table S7, Fig. 2C). To better understand these effects, we conducted post hoc *t*-tests to compare the participants with a high F/B ratio and a negative change in tyrosine in the placebo group compared to the treatment group. We found a significant difference between placebo and treatment groups (*t*(14.849) = −2.14, *P* = 0.049). Put differently, participants with a low homeostatic balance of the gut microbiome (i.e. high F/B ratio) showed a stronger reduction of their tyrosine levels following the intervention, which translated into an increase in their altruistic punishment behavior.

## Discussion

Previous work has shown correlations between microbiome composition and socio-affective behavior, but causal evidence for gut microbiome effects on human behavior is scarce. We experimentally and temporarily manipulated the gut microbiome composition using a dietary intervention and demonstrated that changes in gut microbiome composition influenced altruistic social punishment in a standard behavioral economics game. Following the dietary intervention, participants became less rational (in the sense of classical rational choice theory in economics (45)) and more sensitive to social considerations (the fairness of a monetary offer). Our findings on social decision-making add to recent studies that used active gut microbiome manipulations in humans to show effects on other higher cognitive functions, such as cognitive reactivity and emotional states (46, 47), recognition and recall of emotional pictures (48), and risk-taking and making future-oriented choices in a financial decision-making task (49).

Our work has implications for several research areas: Previous findings suggest that altruistic punishment behavior can be changed by what we eat (34). Since one prominent modulator of the gut microbiome is the host's diet (35, 36), it is plausible that the gut microbiome composition might impact altruistic punishment, not only in an acute phase (after a single meal) but also in the longer term. Previous work has focused on the direct link between food intake and the immediate impact on altruistic punishment. Here, with a subtle dietary intervention over the course of 7 weeks, we demonstrated for the first time (to the best of our knowledge) a causal effect of changing the gut microbiome on altruistic punishment behavior. These effects depended on the baseline intestinal homeostasis of the participants: The “unhealthier” the ratio of the two most common phyla of bacteria (i.e. the higher the F/B ratio), the more our intervention changed the gut microbiome composition and increased altruistic punishment behavior.

What are the potential physiological mechanisms of these effects? Previous work has suggested that diets leading to a decrease in dopamine precursors result in increased altruistic punishments (34). Our findings are in line with this previous work but show that changes in microbiome composition might potentially also affect dopamine-precursor availability independent of dietary intake of these precursors. Our work suggests that the microbiome is both a potential mediator of previously demonstrated dietary effects on behavior and a diet-independent contributor to dopamine-precursor (i.e. tyrosine) availability. Similar to previous work that investigated subtle, diet-induced changes in altruistic punishment, we did not find any significant intervention effect on tryptophan (34). Yet tryptophan and serotonin have been related to social decision-making in other studies (31, 33), and more research in animals and humans is needed to better understand the links between neurotransmitter systems, gut microbiome composition, and changes in social decision-making. An important consideration when interpreting our results is that LNAAs do not directly cross the blood–brain barrier, but their transport is facilitated by the large neutral amino acid transporter 1 (LAT1). Based on the previous literature (30, 34, 50), we controlled for the competition between different large amino acids on LAT1 by computing the ratio of tyrosine to LNAA levels in the blood. However, exactly how blood tyrosine levels translate to brain tyrosine levels and to dopaminergic activity remains an unresolved question for future research.

Our findings show that pre-existing differences in the composition and homeostasis of the gut microbiome (i.e. the F/B ratio), which might reflect habitual dietary patterns and other physiological and environmental factors, modulated the effects of the synbiotic intervention on social decision-making behavior. This is in line with the broader idea that the effects of interventions targeted at the gut microbiome may strongly depend on the gut microbiome composition at baseline and on other individual factors (51). We call for future research to further dissect these kinds of interactions and to link individual malleability of the gut microbiome and social behavior to more stable personality factors and genetic dispositions.

Diet has been previously found to impact the composition of the microbiome differently in males and females (52). Moreover, the microbiome might interact with sex chromosome complement and gonadal hormones, influencing metabolism in distinct ways for males and females (53). Given that our study is a first proof of concept, we collected data only from male participants, which allowed us to exclude potential influences related to the female menstrual cycle on metabolites (54). While this limits the generalizability of our findings, it allowed us to establish initial evidence of gut microbiome effects on social decision-making before expanding to a more diverse sample. Similarly, our inclusion criteria consisted of having a BMI between 20 and 34 and not adhering to a special diet such as vegan, gluten-free, or allergy-related. Future studies could test the effects in more diverse samples and across different diets and existing health conditions. We also note that we used a version of the UG in which all participants had the role of responder. Future studies could also investigate gut microbiome effects on the proposer and other versions of the UG as well as on other social decision-making tasks.

Previous research has suggested that the gut microbiome is altered in clinical conditions linked to socio-affective processes such as depression, anxiety, and autism (8, 14, 15), but the evidence was mostly correlational. Here, we showed that experimental manipulation of the microbiome composition changed social decision-making in healthy male participants, pointing at a causal and broader role of the microbiome for social cognition and behavior, which are typically altered in the clinical conditions mentioned above. Future studies could test whether the gut microbiome could serve as a target for interventions to improve social decision-making in health and disease.

In conclusion, our findings challenge the classical view in cognitive sciences that complex behaviors such as social decision-making are only a function of higher cognitive processes located in cortical brain areas. Instead, our findings suggest that social decision-making is influenced not only by our brain but also by the microorganisms that inhabit our gut and by other bodily factors.

## Materials and methods

The ethics committee of the University Hospital Bonn in Germany, where the data were collected, approved this study (number: 347/18). All participants gave written informed consent according to the Declaration of Helsinki and received monetary compensation for their participation. The study design and analysis plan were preregistered at Open Science Framework (OSF; https://osf.io/utsn4) prior to the start of the data collection. A detailed description of any deviations from the preregistration and the reasons for the deviations can be found below. Any exploratory analyses that we did not preregister are labeled as such. All materials, the data, and analysis scripts are available on OSF (https://osf.io/nk2mb/?view_only=8a28966796614333b3234af923b1ed63, https://osf.io/t36b5/?view_only=9aaf5a7848b44c4084d67a19b2a07a18).

### Participants

One hundred seventeen male participants were recruited at a German university. Data exclusion criteria were preregistered, and 16 participants had to be excluded due to antibiotic treatment (*n* = 8), other changes in medical conditions (e.g. gastroenteritis, *n* = 3), or treatments that had the potential to impact gut microbiome composition/blood parameters (intake of other medication or drastic changes in diet, *n* = 4), or because they did not attend the second session (*n* = 1). Thus, subsequent data analysis was done on 101 male participants (age: 20.3–60.2, M = 32.05, SD = 10.59).

### Study set-up

We conducted a randomized, double-blind, placebo-controlled study consisting of two identical sessions carried out about 7 weeks apart for each participant. Before each session, participants filled in a 3-day food diary. Participants arrived in the lab in a fasted state. Upon arrival in the lab, participants handed over their fecal samples (analysis described below). After blood samples were drawn, participants received a sandwich (451–479 kcal) and hot beverage as breakfast. For the following 3 h, participants received no further food.

During each session, participants undertook various behavioral tasks, including the UG described in detail below. Further, data were collected through a physical exam (i.e. blood pressure, height, weight, body fat, and water). Moreover, during each session, peripheral physiological and brain measures were acquired (task-based functional MRI and diffusion-weighted imaging). Some of the results from these other tasks and measures have been recently published (55), and other results will be reported elsewhere.

After the first session, participants were randomly allocated to either a treatment or a placebo group; the groups received different dietary supplements that they were instructed to take in addition to their diet, starting after the random group assignment and after the first session. Thus, we used a mixed 2 × 2 factorial design with the between-participants factor group (treatment vs. placebo) and within-participants factor session (before vs. after the intake of the dietary supplement). Previous work (56) has observed significant shifts in the microbiome following substantial dietary changes, so we opted for a relatively long, 7-week intervention to maximize the potential effects of our rather small dosage intervention within the limits of study feasibility (MRI scanner availability and potential attrition of participants [57]).

### Dietary supplement

We used a commercially available dietary supplement provided by the manufacturer MensSana. The product, Biotic Junior, contains a total of 2 × 10^9^ colony-forming units per dose consisting of the probiotics *Bifidobacterium lactis*, *Lactobacillus animalis*, *Lactobacillus casei*, *Lactobacillus salivarius*, and *Lactococcus lactis* and the prebiotic inulin (information on the product can be found here: https://osf.io/fbwp7?view_only=8a28966796614333b3234af923b1ed63). The supplement manufacturer provided us with an identical-looking placebo consisting of microcrystalline cellulose, which does not have an effect on microbiome composition.

A similar approach of using a commercially available supplement has been used in previous research, for instance to test the effects of a probiotic intervention on risk and time preferences (49) or by using fermented milk products to explore influences on brain activity (58). To the best of our knowledge, only one previous study has used a commercially available product and then measured the impact of the manipulation on the microbiome. In this study, participants were randomly assigned to either a probiotic, placebo, or no-intervention group. They found a subtle change in the microbiome following the probiotic intervention that was related to performance in an emotional recognition memory task (48).

### UG task

The UG (18) is a task from behavioral economics in which two players (one proposer and one responder) have to share a sum of money. It aims at exploring how people make economic decisions about accepting and receiving money while interacting with others. The proposer is endowed with a sum of money and decides how much of it to offer to the responder. The responder then either rejects the offer, in which case neither player receives any money, or accepts, in which case the responder receives the amount the proposer offered while the proposer keeps the rest of the sum. In our version of this game, all participants had the role of responder and had to decide whether to accept or reject varying offers ranging from €0 to €5 out of €10 (see Table S7). In total there were 20 repeated one-shot trials, meaning participants thought they would never be paired with the same proposer.

### Fecal samples

Stool samples were taken before and after the intervention, with participants bringing a sample of their stool collected in a test tube to both experimental sessions. Samples were stored by the participants for a maximum of 24 h at room temperature before being frozen at −80°C upon arrival (detailed instructions are shared on OSF here: https://osf.io/nk2mb/?view_only=8a28966796614333b3234af923b1ed63). We used the QIAamp PowerFecal Pro DNA Kit to isolate DNA from fecal samples (59). The composition of the microbiome was assessed using 16S rRNA metagenomics. The primer combination of 341f-806bR was used for the gene sequencing of the V3–V4 regions of collected fecal samples. This allowed the characterization of alpha- and beta-diversity as well as the bacterial composition on different taxonomic levels. Thus, we were able to retrieve information on relative abundances from phylum down to species level. Notably, the most abundant phyla were Firmicutes and Bacteroidetes. We used rarefaction, a technique for the estimation of the microbial richness of the sample. More specifically, we used QIIME 2 for the diversity analyses of the microbiome (60). Using this rarefaction technique, we defined based on a feature table the sampling depth of 42,251 sequences as a threshold. Samples below this threshold were excluded. This led to the exclusion of 21 participants for diversity analyses (10 in the treatment group and 11 in the placebo group). Because our primary goal was to investigate changes in microbiome composition over time, we focused on the beta-diversity, which describes differences in microbiome composition between samples, rather than alpha diversity, which reflects the diversity of the microbiome within each sample (13).

### Blood samples

Samples of around 30 mL of blood were taken from participants upon arrival at the lab in the morning (after an overnight fasting period of 12 h). Centrifugation of blood samples was done for 10 min at 3,000 g at room temperature. The samples were then aliquoted and stored at −80°C. Blood samples were of interest notably to assess LNAAs. The LNAAs that were sampled in this study were phenylalanine, leucine, threonine, tyrosine, tryptophan, isoleucine, valine, methionine, and histidine. LNAAs cross the blood–brain barrier using the same LNAA carrier, making it important to consider the blood ratios of LNAAs of interest to estimate the uptake in the brain (50). Therefore, we built ratios for all LNAAs by dividing the amino acid of interest by the sum of the remaining LNAAs. For example,


$$\begin{array}{rl} \mathrm{Tyrosine}/\mathrm{LNAA}\;\mathrm{ratio}\;\;= & \mathrm{Tyrosine}/(\mathrm{Tryptophan}+\mathrm{Phenylalanine}\\ & +\;\mathrm{Leucine}+\mathrm{Isoleucine}+\mathrm{Valine}\\ & +\mathrm{Threonine}+\mathrm{Methionine}\;+\;\mathrm{Histidine}) \end{array}$$

As part of the computation of a metabolic health index (see below), we further sampled the following blood parameters: glucose, insulin, alanine transaminase, aspartate aminotransferase, fasting serum triglycerides, cholesterol, creatine, and C-reactive protein. The analysis of blood parameters followed Roche/Hitachi cobas c systems (Roche Diagnostics, Mannheim, Germany). In particular, blood analyses were carried out according to the hexokinase method (glucose), by electrochemiluminescence immunoassay (insulin), by photometric assay (alanine transaminase and aspartate aminotransferase), by enzymatic colorimetric assay (fasting serum triglycerides and cholesterol), by spectrophotometric assay (creatine), and by particle-enhanced turbidimetric immunoassay (C-reactive protein).

### Metabolic health score

We computed a metabolic health score that gives a more nuanced picture of participants’ metabolic health. We combined BMI, systolic and diastolic blood pressure, insulin, creatine, body fat, alanine transaminase, aspartate aminotransferase, fasting serum triglycerides, cholesterol, C-reactive protein, and the homeostatic model assessment for insulin resistance. We used reference values for each of the parameters and scored participants accordingly. If a particular value was within the range of what is considered healthy, it was scored 1; if the value ranked within what is classified as high-risk, it received 2 points; and if it fell in the very high-risk category, it was scored 3. The sum of all values defined the metabolic health score for each participant. High values reflect the risk of having lower metabolic health. Values in our sample range from 13 to 31 (M = 17.12, SD = 3.66).

### Data analysis

All data were analyzed with R (RStudio 2021.09.0). All numerical values were normalized to account for different measurement scales. We controlled our models for BMI, age, and metabolic health in additional regression models detailed in the respective tables.

We ran the following statistical models:

1) To test whether a change in the gut microbiome composition changed altruistic social punishments, we used generalized linear mixed-effects models on a trial level to test for differences across sessions and between groups. Our dependent variables were the responder's accept vs. reject decisions in all trials of both sessions. Our generalized linear mixed-effects models had “session” as a repeated factor. To account for individual differences, we included a random effect for each participant. We included another random effect for trials. We entered group, session, and their interactions as fixed effects into the model (model 1.1). We repeated the same analysis controlling for age and BMI at baseline (model 1.2) and for age and metabolic health score (model 1.3).
2) Since we observed baseline group differences in rejection behavior,
  a. we calculated Wilcoxon signed-rank tests to see whether changes in rejection rates were different from zero for treatment and placebo groups.
  b. to test whether a change in rejection of unfair offers was significantly different for the groups, we calculated the difference in rejection of unfair offers between sessions and then compared the groups using a linear regression.
3) To investigate whether a 7-week treatment with a synbiotic dietary supplement, as compared to a placebo treatment, increased the diversity of the gut microbiome in human participants, we used linear models with beta-diversity (a measure of difference between samples) as the dependent variable and group, F/B ratio, and their interaction as independent variables (model 2.1). We further controlled for age and BMI (model 2.2) and age and metabolic health score (model 2.3).
4) To investigate whether the relationship between the intake of a supplement and changes in rejection behavior was also moderated by the F/B ratio, we used a generalized linear mixed-effects model, looking at trial levels. Our dependent variable was all trials of both sessions. We entered group, F/B ratio, session, and their interactions as fixed effects into the model (model 1.4). We then ran a post hoc *t*-test to compare the rejection behavior of individuals with a higher F/B ratio in the placebo vs. treatment groups (see Supplementary analyses).
5) Subsequently, we explored underlying mechanistic links of our results by testing through linear models whether our intervention had, depending on the baseline F/B ratio, effects on the changes in LNAA concentrations. Specifically, we ran linear models for each LNAA as the dependent variable with group and F/B ratio as independent variables (model 3.1). These analyses were repeated controlling for age and BMI at baseline (model 3.2) and for age and metabolic health score (model 3.3). For the significant LNAA (tyrosine), we then used a median split to categorize our participants into negative and positive changes in tyrosine/LNAA. Finally, to explore whether these potential changes in LNAA concentrations are also predictive of rejection behavior, we ran a linear model. Group, F/B ratio, and the change in tyrosine/LNAA (categorical based on median split) and all interactions were our independent variables, while the changes in rejection behavior were the dependent variable. Finally, we ran post hoc *t*-tests comparing individuals with a high F/B ratio in the placebo and treatment groups (see Supplementary analyses S1 and S2).

### Deviations from the preregistered analyses

We implemented several analyses that we did not preregister:

1) The unexpected finding that our intervention effect on gut microbiome beta-diversity depended on the ratio of the two most abundant phyla (Firmicutes and Bacteroidetes) led us to include this ratio in the analyses with rejection rates as the dependent variable.
2) We preregistered to explore in a secondary analysis whether changes in tryptophan before and after the intervention could be linked to changes in rejection rates. Given that different LNAAs cross the blood–brain barrier, we decided to look at all LNAAs and then focus in further analysis on those that were impacted by our intervention.

## Supplementary Material

pgae166_Supplementary_Data

## Data Availability

The study was preregistered at Open Science Framework (OSF; https://osf.io/utsn4). Materials, data, and analysis scripts are available on OSF (materials: https://osf.io/nk2mb/?view_only=8a28966796614333b3234af923b1ed63; data and analysis scripts: https://osf.io/t36b5/?view_only=9aaf5a7848b44c4084d67a19b2a07a18).

## Notes

a Except for a change in vitamin C intake for participants in the placebo group. Controlling for this change did not change our results (see Supplementary material—Vitamin C).

b Other behavioral outcomes and their neural correlates have been studied and reported elsewhere. A detailed overview can be found on OSF (https://osf.io/nk2mb/?view_only=8a28966796614333b3234af923b1ed63).
