# Supplementary material for: Impact of the gut microbiome composition on social decision-making
Source: PNAS Nexus. 2024 May 14;3(5):pgae166. doi: 10.1093/pnasnexus/pgae166 (PMC11093127; doi:10.1093/pnasnexus/pgae166)
Supplement: pgae166_Supplementary_Data [file pgae166_supplementary_data.zip › R2_PNAS_Nexus_SI_resubmission_final.docx]

**
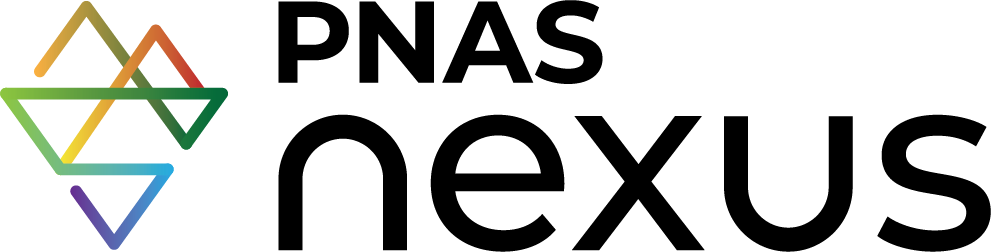
**

**Supplementary Information for**

Impact of the Gut Microbiome Composition on Social Decision-Making

Marie Falkenstein, Marie-Christine Simon, Aakash Mantri, Bernd Weber, Leonie Koban, and Hilke Plassmann

Corresponding author: Hilke Plassmann

Email: hilke.plassmann@insead.edu

**This PDF file includes:**

Supplemental Analyses 1.1 and 1.2

Tables S1 to S7

# Supplemental Analyses

## **Vitamin C — analysis**

We repeated the analyses presented in Supplementary Table S3 controlling for the change in vitamin C for the first and fourth models. Our results hold when controlling for the change in vitamin C.

In detail, for the model with choice as dependent variable (accept or reject) reported in Table S3, we found the interaction to remain significant when controlling for vitamin C: (β = 1.126, std. error = 0.282, *p* < 0.001).

When including F/B ratio (as in model 4 in Table S3), the three-way interaction also remains significant when controlling for the change in vitamin C (β = 1.366, std. error = 0.237, *p* < 0.001).

## **Post hoc *t*-tests for participants with high F/B ratio**

We conducted post hoc group comparisons to better understand the direction of our interaction effects for the analysis reported in model 4 of Table S3. For participants with a high F/B ratio, we ran a *t*-test with choices (reject/accept) as the dependent variable, and group as predictor. We found a significant difference between the placebo and treatment groups: *t*(1971.7) = 2.73, *p* = 0.006.

We also conducted post hoc group comparisons to better understand the direction of our interaction effects for analysis reported in Table S6. We compared the placebo and treatment groups for participants with a high F/B ratio and a negative change in tyrosine. Across all offers, we found no difference between groups: *t*(11.565) = -1.37, *p* = 0.195. Concentrating on unfair offers, we saw a significant difference between placebo and treatment groups: *t*(14.849) = -2.14, *p* = .049.

# Supplemental Tables

**Supplementary Table S1:** Group Differences Socio- and Psychographics

| Variable | M (SEM) treatment | M (SEM) placebo | Statistic value | *p* value | Test type |
| --- | --- | --- | --- | --- | --- |
| ***Health-Related Variables*** | | | | | |
| Age | 32.80 (-1.58) | 31.27 (1.40) | 1345 | 0.637 | non-parametric |
| BMI | 25.83 (0.44) | 25.55 (0.43) | 1356 | 0.585 | non-parametric |
| Metabolic health parameter | 17.26 (0.55) | 16.98 (0.49) | 1314 | 0.792 | non-parametric |
| Birmingham IBS Symptom Questionnaire | 6.06 (0.64) | 5.56 (0.57) | 1210 | 0.672 | non-parametric |
| Quality of Life Questionnaire: Global quality of life | 11.55 (0.25) | 11.4 (0.25) | 1339 | 0.66 | non-parametric |
| Quality of Life Questionnaire: Physical functioning | 5.67 (0.14) | 5.56 (0.17) | 1373 | 0.44 | non-parametric |
| Quality of Life Questionnaire: Role functioning | 2.71 (0.18) | 2.56 (0.15) | 1326.5 | 0.66 | non-parametric |
| Quality of Life Questionnaire: Emotional functioning | 7.06 (0.27) | 7.04 (0.35) | 1335.5 | 0.68 | non-parametric |
| Quality of Life Questionnaire: Cognitive functioning | 2.92 (0.15) | 2.96 (0.16) | 1260.5 | 0.919 | non-parametric |
| Quality of Life Questionnaire: Social functioning | 2.41 (0.15) | 2.22 (0.08) | 1311 | 0.711 | non-parametric |
| Quality of Life Questionnaire: Fatigue | 5.86 (0.22) | 5.72 (0.24) | 1383.5 | 0.455 | non-parametric |
| Quality of Life Questionnaire: Nausea and vomiting | 2.12 (0.06) | 2.18 (0.06) | 1177 | 0.238 | non-parametric |
| Quality of Life Questionnaire: Pain | 2.84 (0.20) | 2.82 (0.17) | 1222 | 0.687 | non-parametric |
| Quality of Life Questionnaire: Dyspnea | 1.18 (0.07) | 1.1 (0.06) | 1369.5 | 0.218 | non-parametric |
| Quality of Life Questionnaire: Sleep disturbance | 1.49 (0.09) | 1.6 (0.10) | 1181.5 | 0.476 | non-parametric |
| Quality of Life Questionnaire: Appetite loss | 1.29 (0.09) | 1.12 (0.06) | 1425 | 0.109 | non-parametric |
| Quality of Life Questionnaire: Constipation | 1.16 (0.06) | 1.18 (0.07) | 1289.5 | 0.87 | non-parametric |
| Quality of Life Questionnaire: Diarrhea | 1.16 (0.06) | 1.26 (0.08) | 1168 | 0.276 | non-parametric |
| Quality of Life Questionnaire: Financial impact | 1.14 (0.08) | 1.08 (0.04) | 1254 | 0.752 | non-parametric |
| ***Mood, Affect, and Personality*** | | | | | |
| Profile of Mood States: Numbness | 6.31 (0.92) | 7.36 (1.42) | 1324.5 | 0.737 | non-parametric |
| Profile of Mood States: Vigor | 24.78 (0.95) | 23.94 (1.07) | 0.589 | 0.557 | *t*-test |
| Profile of Mood States: Fatigue | 10.33 (1.04) | 9.68 (1.12) | 1361 | 0.561 | non-parametric |
| Profile of Mood States: Irritability | 4.65 (0.68) | 4.86 (0.89) | 1348.5 | 0.616 | non-parametric |
| Positive and Negative Affect Schedule: Positive affect | 21.33 (0.42) | 20.86 (0.48) | 0.744 | 0.459 | *t*-test |
| Positive and Negative Affect Schedule: Negative affect | 20.69 (0.46) | 20.52 (0.48) | 0.248 | 0.804 | *t*-test |
| BFI 2: The Next Big Five Inventory | | | | | |
| BFI 2: Extraversion | 40.20 (1.17) | 41.36 (1.15) | -0.71 | 0.479 | *t*-test |
| BFI 2: Agreeableness | 47.71 (0.64) | 46.18 (0.83) | 1479.5 | 0.165 | non-parametric |
| BFI 2: Conscientiousness | 42.65 (0.82) | 42.88 (1.02) | -0.179 | 0.859 | *t*-test |
| BFI 2: Negative emotionality | 26.37 (0.92) | 27.26 (1.09) | -0.623 | 0.535 | *t*-test |
| BFI 2: Open-mindedness | 44.51 (1.06) | 44.58 (1.11) | -0.046 | 0.964 | *t*-test |
| BFI 2: Sociability | 12.84 (0.47) | 13.3 (0.50) | -0.662 | 0.51 | *t*-test |
| BFI 2: Assertiveness | 13.20 (0.46) | 13.74 (0.47) | 1157.5 | 0.425 | non-parametric |
| BFI 2: Energy level | 14.16 (0.39) | 14.32 (0.43) | 1268.5 | 0.967 | non-parametric |
| BFI 2: Compassion | 16.37 (0.29) | 15.42 (0.41) | 1542.5 | 0.066 | non-parametric |
| BFI 2: Respectfulness | 17.22 (0.25) | 16.98 (0.27) | 1353.5 | 0.588 | non-parametric |
| BFI 2: Trust | 14.12 (0.31) | 13.78 (0.37) | 1341 | 0.653 | non-parametric |
| BFI 2: Organization | 14.59 (0.38) | 14.3 (0.47) | 1338 | 0.67 | non-parametric |
| BFI 2: Productiveness | 12.88 (0.40) | 13.42 (0.37) | -0.993 | 0.323 | *t*-test |
| BFI 2: Responsibility | 15.18 (0.31) | 15.16 (0.36) | 1252 | 0.877 | non-parametric |
| BFI 2: Anxiety | 10 (0.38) | 9.98 (0.45) | 0.034 | 0.973 | *t*-test |
| BFI 2: Depression | 7.65 (0.32) | 7.8 (0.36) | 1253 | 0.883 | non-parametric |
| BFI 2: Emotional volatility | 8.73 (0.38) | 9.48 (0.45) | 1098.5 | 0.229 | non-parametric |
| BFI 2: Aesthetic sensitivity | 13.86 (0.52) | 13.34 (0.62) | 1312 | 0.803 | non-parametric |
| BFI 2: Intellectual curiosity | 15.73 (0.37) | 16.18 (0.42) | 1102.5 | 0.239 | non-parametric |
| BFI 2: Creative imagination | 14.92 (0.43) | 15.06 (0.34) | 1263.5 | 0.94 | non-parametric |
| The State-Trait Anxiety Inventory: Trait | 28.33 (1.47) | 29.74 (1.67) | -0.634 | 0.527 | *t*-test |
| The State-Trait Anxiety Inventory: State | 28.20 (1.27) | 30.4 (1.53) | -1.111 | 0.269 | *t*-test |
| The Impulsive Behavior Short Scale (I-8): Urgency | 5.37 (0.25) | 5.52 (0.23) | 1214 | 0.676 | non-parametric |
| The Impulsive Behavior Short Scale (I-8): Lack of premeditation | 7.78 (0.18) | 7.78 (0.21) | 1250 | 0.862 | non-parametric |
| The Impulsive Behavior Short Scale (I-8): Lack of perseverance | 7.18 (0.18) | 7.54 (0.21) | 1063.5 | 0.141 | non-parametric |
| The Impulsive Behavior Short Scale (I-8): Sensation seeking | 6.55 (0.19) | 7.16 (0.28) | 1052.5 | 0.125 | non-parametric |
| Perceived Stress Questionnaire | 55.27 (1.61) | 55.8 (2.2) | 1332 | 0.701 | non-parametric |
| ***Food Journals and Eating Behavior*** | | | | | |
| Three-Factor Eating Questionnaire: Uncontrolled eating | 5.82 (0.54) | 4.96 (0.50) | 1433 | 0.283 | non-parametric |
| Three-Factor Eating Questionnaire: Cognitive restraint | 5.45 (0.38) | 4.98 (0.38) | 1364.5 | 0.541 | non-parametric |
| Three-Factor Eating Questionnaire: Emotional eating | 4.84 (0.43) | 4.7 (0.45) | 1326 | 0.73 | non-parametric |
| Yale Food Addiction Scale: Amount | 0.06 (0.03) | 0.12 (0.11) | 1221 | 0.437 | non-parametric |
| Yale Food Addiction Scale: Attempts | 0.22 (0.07) | 0.36 (0.11) | 1189 | 0.426 | non-parametric |
| Yale Food Addiction Scale: Time | 0.02 (0.02) | 0.04 (0.03) | 1249 | 0.556 | non-parametric |
| Yale Food Addiction Scale: Activities | 0 (0) | 0 (0) | N/A | N/A | N/A |
| Yale Food Addiction Scale: Problems | 0.06 (0.03) | 0.02 (0.02) | 1324.5 | 0.325 | non-parametric |
| Yale Food Addiction Scale: Withdrawal | 0.08 (0.05) | 0.12 (0.06) | 1223.5 | 0.459 | non-parametric |
| Yale Food Addiction Scale: Impairment/distress | 0 (0) | 0 (0) | N/A | N/A | N/A |
| Yale Food Addiction Scale: Obligations | 0.04 (0.03) | 0.02 (0.02) | 1299.5 | 0.579 | non-parametric |
| Yale Food Addiction Scale: Consequences | 0.02 (0.02) | 0.12 (0.06) | 1172 | 0.089 | non-parametric |
| Yale Food Addiction Scale: Tolerance | 0.02 (0.02) | 0.04 (0.03) | 1249 | 0.556 | non-parametric |
| Yale Food Addiction Scale: Situations | 0.04 (0.03) | 0.04 (0.03) | 1274 | 0.992 | non-parametric |
| Yale Food Addiction Scale: Craving | 0.04 (0.03) | 0.1 (0.04) | 1197.5 | 0.234 | non-parametric |
| Energy in kcal | 2786.03 (82.14) | 2897.26 (100.05) | 1179 | 0.517 | non-parametric |
| Water | 1728.67 (86.99) | 1696.898 (82.86) | 0.264 | 0.792 | *t*-test |
| Protein | 112.69 (6.07) | 113.8 (4.71) | 1153 | 0.409 | non-parametric |
| Protein (percentage) | 16.45 (0.65) | 16.2 (0.52) | 1243.5 | 0.832 | non-parametric |
| Fat | 117.19 (3.41) | 122.86 (5.50) | 1221.5 | 0.719 | non-parametric |
| Fat (percentage) | 37.98 (0.84) | 37.72 (1.01) | 0.198 | 0.844 | *t*-test |
| Carbohydrates | 294.75 (12.48) | 313.18 (15.43) | 1155 | 0.417 | non-parametric |
| Carbohydrates (percentage) | 43.06 (1.03) | 44.24 (1.10) | -0.786 | 0.434 | *t*-test |
| Fiber | 24.19 (1.06) | 28.31 (3.05) | 1167.5 | 0.467 | non-parametric |
| Alcohol | 10.85 (2.66) | 7.77 (1.70) | 1330 | 0.708 | non-parametric |
| Alcohol (percentage) | 2.57 (0.61) | 1.84 (0.41) | 1349 | 0.59 | non-parametric |
| Polyunsaturated fatty acids | 16.73 (0.89) | 18.09 (0.95) | 1118 | 0.288 | non-parametric |
| Cholesterol | 479.20 (35.01) | 411.16 (29.10) | 1442 | 0.258 | non-parametric |
| Vitamin A | 1516.87 (96.50) | 1600.97 (130.21) | 1255 | 0.895 | non-parametric |
| Carotene | 4.79 (0.48) | 5.58 (0.76) | 1281.5 | 0.967 | non-parametric |
| Vitamin E | 14.61 (0.72) | 15.54 (0.90) | 1187.5 | 0.555 | non-parametric |
| *Vitamin B1* | *1.47 (0.07)* | *1.81 (0.15)* | *952.5* | *0.029* | *non-parametric* |
| Vitamin B2 | 1.94 (0.12) | 1.91 (0.09) | 1199.5 | 0.61 | non-parametric |
| Vitamin B6 | 2.08 (0.10) | 2.28 (0.11) | -1.339 | 0.184 | *t*-test |
| Folic acid | 307.45 (11.82) | 322.51 (20.90) | 1318 | 0.773 | non-parametric |
| Vitamin C | 117.90 (9.30) | 148.85 (18.05) | 1133 | 0.336 | non-parametric |
| Sodium | 3052.31 (159.02) | 3224.44 (168.56) | -0.743 | 0.459 | *t*-test |
| Potassium | 3616.89 (131.64) | 4029.52 (213.91) | 1085 | 0.198 | non-parametric |
| Calcium | 1065.96 (51.04) | 1056.70 (63.90) | 1352 | 0.603 | non-parametric |
| Magnesium | 403.11 (13.41) | 447.96 (32.54) | 1115.5 | 0.28 | non-parametric |
| Phosphorus | 1811.34 (60.26) | 1849.27 (94.08) | 1262 | 0.932 | non-parametric |
| Iron | 15.00 (0.53) | 16.53 (1.33) | 1258.5 | 0.913 | non-parametric |
| Carbohydrates/protein ratio | 2.81 (0.12) | 2.88 (0.12) | -0.385 | 0.701 | *t*-test |
| ***Gut Microbiome Characteristics*** | | | | | |
| GMB alpha diversity (Shannon) | 6.55 (0.11) | 6.53 (0.07) | 1077 | 0.743 | non-parametric |
| GMB alpha diversity (Faith) | 35.3 (1.41) | 34.2 (1.18) | 0.615 | 0.540 | *t*-test |
| F/B ratio | 1.70 (0.43) | 1.20 (0.13) | 1159 | 0.433 | non-parametric |
| **χ2 tests, demographic variables** | | | | | |
|  | df | Median treatment | Median placebo | χ-squared | *p* value |
| Income: What is your annual household income? | 11 | €40.000–49.999 | €10.000–19.999 | 12.344 | .338 |
| Education | 8 | Bachelor’s degree | Diploma | 4.283 | .831 |
| Sleep: How many hours do you sleep on an average night? | 2 | 6–8 hours | 6–8 hours | 1.312 | .519 |
| Sleep: Do you feel like you get enough sleep? | 1 | Yes | Yes | 0.877 | .349 |
| Exercise: How many hours per week do you exercise on average? | 6 | 2–4 hours | 2–4 hours | 6.379 | .382 |
| Exercise: How intense is your physical activity on average? | 3 | Intensive | Intensive | 2.177 | .536 |

**Supplementary Table S2:** Changes Over Session in Diet and Physiology

| Variable | M (SEM) treatment | M (SEM) placebo | Statistic value | *p* value | Test type |
| --- | --- | --- | --- | --- | --- |
| BMI | -0.03 (0.07) | 0.05 (0.00) | 1175 | 0.499 | non-parametric |
| Energy (kcal) | -264.80 (98.15) | -284.05 (92.77) | 0.143 | 0.887 | *t*-test |
| Water | -20.82 (82.44) | -142.85 (80.89) | 1.057 | 0.293 | *t*-test |
| Protein | -12.9 (5.68 ) | -13.8 (4.52) | 1335 | 0.686 | non-parametric |
| Fat | -12.8 (5.26) | -13.2 (5.63) | 0.049 | 0.961 | *t*-test |
| Carbohydrates | -27.66 (13.14) | -35.14 (9.81) | 0.456 | 0.649 | *t*-test |
| Fiber | -1.48 (1.50) | -3.89 (1.17) | 1371 | 0.517 | non-parametric |
| Alcohol | 1.82 (2.49) | 4.98 (2.32) | 1257 | 0.905 | non-parametric |
| Polyunsaturated fatty acids | 0.26 (1.10) | 0.03 (1.20) | 0.137 | 0.891 | *t*-test |
| Cholesterol | -64.20 (39.4) | -17.58 (35.11) | 1179 | 0.517 | non-parametric |
| Vitamin A | -162.54 (146.23) | -351.70 (120.52) | 1354 | 0.594 | non-parametric |
| Vitamin E | -0.13 (0.93) | -1.53 (0.93) | 1.064 | 0.29 | *t*-test |
| Vitamin B1 | 0.03 (0.10) | -0.25 (0.09) | 1514.5 | 0.104 | non-parametric |
| Vitamin B2 | -0.22 (0.11) | -0.25 (0.09) | 1276.5 | 0.995 | non-parametric |
| Vitamin B6 | -0.08 (0.13) | -0.35 (0.12) | 1.55 | 0.124 | *t*-test |
| Folic acid | -22.15 (16.52) | -41.82 (16.21) | 1362 | 0.557 | non-parametric |
| *Vitamin C* | *12.84 (12.87)* | *-36.92 (17.04)* | *1611* | *0.023* | *non-parametric* |
| Sodium | -298.12 (177.10) | -291.33 (176.86) | 1181 | 0.525 | non-parametric |
| Potassium | -247.12 (164.06) | -684.31 (158.20) | 1.918 | 0.058 | *t*-test |
| Calcium | -65.49 (70.02) | -29.39 (61.70) | 1121 | 0.297 | non-parametric |
| Magnesium | -23.33 (16.10) | -60.94 (16.98) | 1.608 | 0.111 | *t*-test |
| Phosphorus | -146.81 (72.79) | -157.04 (69.47) | 0.102 | 0.919 | *t*-test |
| Iron | -1.13 (0.60) | -2.37 (0.75) | 1.291 | 0.2 | *t*-test |
| Carbohydrates/protein ratio | 0.02 (0.14) | 0.09 (0.14) | 1220 | 0.711 | non-parametric |

**Supplementary Table S3:** Generalized Linear Model Testing Rejection Behavior of All Offers Across Sessions

|  | | | | | | | | | |  |  |  |
| --- | --- | --- | --- | --- | --- | --- | --- | --- | --- | --- | --- | --- |
|  | **Model 1** | | | **Model 2** | | | **Model 3** | | | **Model 4** | | |
| *Predictors* | *Odds ratios* | *CI* | *p* | *Odds ratios* | *CI* | *p* | *Odds ratios* | *CI* | *p* | *Odds ratios* | *CI* | *p* |
| (Intercept) | 0.41 | 0.03 – 6.27 | 0.525 | 0.40 | 0.03 – 6.04 | 0.509 | 0.40 | 0.03 – 6.10 | 0.513 | 0.12 | 0.01 – 2.97 | 0.197 |
| **Group** | **0.16** | **0.03 – 0.72** | **0.017** | **0.17** | **0.04 – 0.76** | **0.021** | **0.16** | **0.04 – 0.75** | **0.020** | 0.42 | 0.05 – 3.97 | 0.452 |
| Session | 0.78 | 0.54 – 1.14 | 0.206 | 0.78 | 0.54 – 1.14 | 0.206 | 0.78 | 0.54 – 1.14 | 0.206 | **4.51** | **2.35 – 8.65** | **<0.001** |
| **Group * Session** | **3.08** | **1.77 – 5.36** | **<0.001** | **3.08** | **1.77 – 5.36** | **<0.001** | **3.08** | **1.77 – 5.36** | **<0.001** | 0.72 | 0.33 – 1.58 | 0.410 |
| F/B ratio |  |  |  |  |  |  |  |  |  | 2.68 | 0.79 – 9.10 | 0.114 |
| Age |  |  |  | 0.76 | 0.35 – 1.63 | 0.476 | 0.71 | 0.31 – 1.61 | 0.412 |  |  |  |
| BMI |  |  |  | 0.77 | 0.36 – 1.65 | 0.496 |  |  |  |  |  |  |
| Metabolic Health Score |  |  |  |  |  |  | 1.00 | 0.44 – 2.24 | 0.991 |  |  |  |
| Group * F/B ratio |  |  |  |  |  |  |  |  |  | 0.39 | 0.11 – 1.40 | 0.149 |
| **Session * F/B ratio** |  |  |  |  |  |  |  |  |  | **0.22** | **0.14 – 0.35** | **<0.001** |
| **Group * Session * F/B ratio** |  |  |  |  |  |  |  |  |  | **3.93** | **2.47 – 6.24** | **<0.001** |
| **Random effects** | | | | | | | | | |  | | |
| σ^2^ | 3.29 | | | 3.29 | | | 3.29 | | | 3.29 | | |
| τ_00_ | 14.17 _ID_ | | | 14.00 _ID_ | | | 14.07 _ID_ | | | 15.31 _ID_ | | |
|  | 32.22 _trial_ | | | 32.23 _trial_ | | | 32.24 _trial_ | | | 35.08 _trial_ | | |
| ICC | 0.93 | | | 0.93 | | | 0.93 | | | 0.94 | | |
| N | 101 _ID_ | | | 101 _ID_ | | | 101 _ID_ | | | 101 | | |
|  | 20 _trial_ | | | 20 _trial_ | | | 20 _trial_ | | | 20 _trial_ | | |
| Observations | 4040 | | | 4040 | | | 4040 | | | 4040 | | |
| Marginal R^2^/ conditional R^2^ | 0.011/0.934 | | | 0.014/0.935 | | | 0.013/0.935 | | | 0.016 / 0.940 | | |

|  | | | |  |  |  |  |  |  |  |  |  |  |
| --- | --- | --- | --- | --- | --- | --- | --- | --- | --- | --- | --- | --- | --- |
| **Supplementary Table S4:** Linear Models Testing Group Differences on Change in Rejection of Unfair Offers | | | | | | | | | | | | |  |
|  | **Model 1 — Change in Rejection** | | | **Model 2 — Change in Rejection** | | | **Model 3 — Change in Rejection** | | | **Model 4 — Change in Rejection** | | |  |
| *Predictors* | *Estimates* | *CI* | *p* | *Estimates* | *CI* | *p* | *Estimates* | *CI* | *p* | *Estimates* | *CI* | *p* |  |
| (Intercept) | -0.22 | -0.49 – 0.06 | 0.125 | -0.22 | -0.50 – 0.05 | 0.112 | -0.22 | -0.50 – 0.05 | 0.114 | -0.32 | -0.60 – -0.05 | **0.022** |  |
| Group | 0.43 | 0.04 – 0.81 | **0.032** | 0.44 | 0.05 – 0.83 | **0.027** | 0.44 | 0.05 – 0.83 | **0.027** | 0.54 | 0.16 – 0.93 | **0.006** |  |
|  |  |  |  | -0.05 | -0.25 – 0.15 | 0.617 |  |  |  |  |  |  |  |
|  |  |  |  | -0.09 | -0.29 – 0.11 | 0.390 | -0.09 | -0.30 – 0.12 | 0.406 |  |  |  |  |
|  |  |  |  |  |  |  | -0.03 | -0.24 – 0.19 | 0.810 |  |  |  |  |
| FB ratio |  |  |  |  |  |  |  |  |  | -0.97 | -1.63 –  -0.31 | **0.005** |  |
| Group × F/B ratio |  |  |  |  |  |  |  |  |  | 0.89 | 0.20 – 1.58 | **0.012** |  |
|  |  |  |  |  |  |  |  |  |  |  |  |  |  |
| Observations | 101 | | | 101 | | | 101 | | | 101 | | |  |
| R^2^ / R^2^ adjusted | 0.046 / 0.036 | | | 0.058 / 0.029 | | | 0.056 / 0.027 | | | 0.128 / 0.101 | | |  |

**Supplementary Table S5:** Linear Models Testing Treatment Effects on the Gut Microbiome Beta Diversity

|  | **Model 1** | | | **Model 2** | | | **Model 3** | | |
| --- | --- | --- | --- | --- | --- | --- | --- | --- | --- |
| *Predictors* | *Estimates* | *CI* | *p* | *Estimates* | *CI* | *p* | *Estimates* | *CI* | *p* |
| (Intercept) | -0.16 | -0.46 – 0.14 | 0.281 | -0.15 | -0.45 – 0.16 | 0.341 | -0.15 | -0.46 – 0.16 | 0.327 |
| F/B ratio | -0.38 | -1.05 – 0.29 | 0.261 | -0.34 | -1.04 – 0.36 | 0.340 | -0.36 | -1.06 – 0.35 | 0.315 |
| Group | 0.21 | -0.20 – 0.63 | 0.302 | 0.21 | -0.21 – 0.63 | 0.323 | 0.21 | -0.21 – 0.63 | 0.332 |
| **F/B ratio * Group** | 0.78 | 0.08 – 1.48 | **0.029** | 0.73 | 0.00 – 1.46 | **0.050** | 0.75 | 0.01 – 1.49 | **0.046** |
| BMI |  |  |  | -0.06 | -0.29 – 0.18 | 0.633 |  |  |  |
| Age |  |  |  | 0.04 | -0.18 – 0.26 | 0.717 | 0.03 | -0.20 – 0.27 | 0.778 |
| Metabolic health score |  |  |  |  |  |  | -0.03 | -0.26 – 0.20 | 0.820 |
| Observations | 80 | | | 80 | | | 80 | | |
| R^2^ / R^2^ adjusted | 0.206 / 0.175 | | | 0.209 / 0.156 | | | 0.207 / 0.154 | | |

**Supplementary Table S6:** Linear Models Testing Treatment Effects on the Change in Large Neutral Amino Acids

| **Change — Tryptophan** | | | | | | | | | | | | | | | | | |
| --- | --- | --- | --- | --- | --- | --- | --- | --- | --- | --- | --- | --- | --- | --- | --- | --- | --- |
|  | | | | **Model 1** | | | | | **Model 2** | | | | | **Model 3** | | | |
| *Predictors* | *Estimates* | | *CI* | | | *p* | *Estimates* | | *CI* | | *p* | *Estimates* | | | *CI* | | *p* |
| (Intercept) | 0.10 | | -0.19 – 0.39 | | | 0.510 | 0.09 | | -0.20 – 0.38 | | 0.550 | 0.09 | | | -0.21 – 0.38 | | 0.554 |
| Group | -0.18 | | -0.58 – 0.22 | | | 0.377 | -0.17 | | -0.57 – 0.24 | | 0.421 | -0.16 | | | -0.57 – 0.24 | | 0.423 |
| F/B ratio | -0.05 | | -0.75 – 0.64 | | | 0.882 | -0.06 | | -0.77 – 0.66 | | 0.871 | -0.07 | | | -0.79 – 0.65 | | 0.851 |
| Group x F/B ratio | -0.11 | | -0.84 – 0.62 | | | 0.768 | -0.10 | | -0.84 – 0.65 | | 0.800 | -0.08 | | | -0.84 – 0.67 | | 0.824 |
| Age |  | |  | | |  | -0.10 | | -0.31 – 0.11 | | 0.332 | -0.11 | | | -0.33 – 0.11 | | 0.340 |
| BMI |  | |  | | |  | -0.00 | | -0.21 – 0.21 | | 0.974 |  | | |  | |  |
| Metabolic health score |  | |  | | |  |  | |  | |  | 0.01 | | | -0.21 – 0.23 | | 0.931 |
| Observations | | | | 101 | | | | | 101 | | | | | 101 | | | |
| R^2^ / R^2^ adjusted | | | | 0.034 / 0.004 | | | | | 0.045 / -0.006 | | | | | 0.045 / -0.006 | | | |
| **Change — tyrosine** | |  | | |  | | |  | |  | | |  | | |  | |
|  | | | | **Model 1** | | | | | **Model 2** | | | | | **Model 3** | | | |
| *Predictors* | *Estimates* | | *CI* | | | *p* | *Estimates* | | *CI* | | *p* | *Estimates* | | | *CI* | | *p* |
| (Intercept) | -0.00 | | -0.29 – 0.28 | | | 0.990 | 0.01 | | -0.27 – 0.29 | | 0.943 | 0.01 | | | -0.27 – 0.30 | | 0.917 |
| Group | 0.09 | | -0.30 – 0.49 | | | 0.642 | 0.09 | | -0.30 – 0.47 | | 0.660 | 0.08 | | | -0.31 – 0.47 | | 0.690 |
| **F/B ratio** | **0.60** | | **-0.09 – 1.28** | | | **0.086** | **0.75** | | **0.07 – 1.44** | | **0.031** | **0.75** | | | **0.06 – 1.45** | | **0.034** |
| **Group x F/B ratio** | -0.81 | | -1.52 – -0.10 | | | **0.026** | -0.97 | | -1.69 – -0.26 | | **0.008** | -0.99 | | | -1.72 – -0.26 | | **0.009** |
| Age |  | |  | | |  | 0.07 | | -0.13 – 0.26 | | 0.509 | 0.09 | | | -0.12 – 0.31 | | 0.388 |
| BMI |  | |  | | |  | -0.23 | | -0.43 – -0.03 | | **0.024** |  | | |  | |  |
| Metabolic health score |  | |  | | |  |  | |  | |  | -0.21 | | | -0.42 – 0.01 | | 0.063 |
| Observations | | | | 101 | | | | | 101 | | | | | 101 | | | |
| R^2^ / R^2^ adjusted | | | | 0.075 / 0.047 | | | | | 0.124 / 0.078 | | | | | 0.109 / 0.062 | | | |

*Note:* There is a significant effect of BMI on how much tyrosine changed over time across groups. This effect was unexpected, and we call for future research to better understand this finding.

**Supplementary Table S7:** Linear Models Testing Effects of Changes in Tyrosine on Rejection Behavior

|  | **Change in rejection of unfair offers** | | |
| --- | --- | --- | --- |
| *Predictors* | *Estimates* | *CI* | *p* |
| (Intercept) | -0.54 | -1.04 – -0.05 | **0.031** |
| **Group** | **0.90** | **0.27 – 1.53** | **0.006** |
| **FB ratio** | **-1.99** | **-3.52 – -0.46** | **0.011** |
| Tyrosine/LNAA (cat) | 0.20 | -0.44 – 0.84 | 0.533 |
| BMI | 0.01 | -0.19 – 0.21 | 0.932 |
| Age | -0.09 | -0.28 – 0.11 | 0.390 |
| Group * F/B ratio | 1.94 | 0.40 – 3.48 | **0.014** |
| Group * Tyrosine/LNAA (cat) | -0.57 | -1.40 – 0.27 | 0.179 |
| F/B ratio * Tyrosine/LNAA (cat) | 1.27 | -0.43 – 2.97 | 0.140 |
| **Group * F/B ratio * Tyrosine/LNAA (cat)** | **-2.19** | **-4.12 – -0.25** | **0.027** |
| Observations | 101 | | |
| R^2^ / R^2^ adjusted | 0.201 / 0.122 | | |

**Supplementary Table S8:** Offers Presented in the Ultimatum Game

|  | |
| --- | --- |
| Offers | Times presented |
| €0 out of €10 | 1 |
| €1 out of €10 | 3 |
| €2 out of €10 | 3 |
| €3 out of €10 | 3 |
| €4 out of €10 | 4 |
| €5 out of €10 | 6 |
